# The association of COVID-19 vaccine availability with mental health among adults in the United States

**DOI:** 10.3389/fpsyt.2022.970007

**Published:** 2022-08-09

**Authors:** Chan Shen, Lucy Rashiwala, R. Constance Wiener, Patricia A. Findley, Hao Wang, Usha Sambamoorthi

**Affiliations:** ^1^Departments of Surgery and Public Health Sciences, Penn State College of Medicine, Hershey, PA, United States; ^2^Department of Pharmacotherapy, College of Pharmacy, University of North Texas Health Science Center, Fort Worth, TX, United States; ^3^Department of Dental Public Health and Professional Practice, School of Dentistry, West Virginia University, Morgantown, WV, United States; ^4^Rutgers School of Social Work, New Brunswick, NJ, United States; ^5^Department of Emergency Medicine, JPS Health Network, Integrative Emergency Services, Fort Worth, TX, United States

**Keywords:** COVID-19, depression, anxiety, Census pulse survey, vaccine availability

## Abstract

**Objective:**

To assess whether COVID-19 vaccine approval and availability was associated with reduction in the prevalence of depression and anxiety among adults in the United States.

**Methods:**

We adopted cross sectional and quasi-experimental design with mental health measurements before vaccine availability (June 2020, *N* = 68,009) and after vaccine availability (March 2021, *N* = 63,932) using data from Census Pulse Survey. Depression and anxiety were derived from PHQ-2 and GAD-2 questionnaires. We compared rates of depression and anxiety between June 2020 and March 2021. Unadjusted and adjusted analysis with replicate weights were conducted.

**Results:**

Depression prevalence was 25.0% in June 2020 and 24.6% in March 2021; anxiety prevalence was 31.7% in June 2020 and 30.0% in March 2021 in the sample. In adjusted analysis, there were no significant differences in likelihood of depression and anxiety between June 2020 and March 2021.

**Conclusion:**

Depression and anxiety were not significantly different between June 2020 and March 2021, which suggests that the pandemic effect continues to persist even with widespread availability of vaccines.

## Introduction

The year 2020 brought unprecedented situations around the globe. During the COVID-19 pandemic, many households faced isolation, fear, violence, drug abuse, and anxiety. The pandemic has impacted every aspect of society resulting in economic uncertainty, limited interpersonal connections, mortality, drug abuse, and social disruption. Furthermore, America has faced riots, protests, police brutality, and political divisiveness as well (1). In April 2020, the Bureau of Labor Statistics (2020) reported a record-high unemployment rate of 14.7% (2). As of June, 2022, more than one million deaths have been reported due to COVID-19 in the US (3).

Numerous studies in the literature have examined the impact of the COVID-19 pandemic on mental health. Two meta-analyses found that prevalence rates of depression and anxiety increased substantially during the COVID-19 outbreak (4, 5). One of them covered 12 studies with sample sizes ranging from 600 to 7,236 participants, and the pooled prevalence of depression was 25% during the COVID-19 outbreak, which is 7 times higher than the global estimated prevalence of depression of 3.44% in 2017 (4). The other meta-analysis paper covering 14 studies on the prevalence of depression with a sample size of 44,531 people, found an even higher prevalence rate of depression at 33.7% during COVID-19 (5). That study also examined prevalence of anxiety using 17 studies with a sample size of 63,439 and estimated the prevalence rate of anxiety to be also very high at 31.9% during COVID-19 (5).

Several studies have further researched the relationship between mental health during the pandemic and multiple factors such as government role, food insufficiency, housing, and income level (6–8). However, few studies considered the impact of vaccine availability and whether it helps reduce mental stress. One study on chronic kidney disease patients found that COVID-19 vaccination improved anxiety and depression in this special group of patients (9). However, there is significant skepticism about COVID-19 vaccine and it is unknown whether the availability of vaccine improved mental health in the general public.

This study aims to fill the knowledge gap by assessing if COVID-19 vaccine availability was associated with reduction in the prevalence of depression and anxiety among a nationally representative group of adults in the US. We compared the prevalence of depression and anxiety before and after COVID-19 vaccine became available using a nationally representative household survey.

## Methods

### Data source

The data source we used for this study is the Census pulse survey. The Census pulse survey is a nationally representative household survey was designed by the United States Census Bureau in collaboration with numerous federal agencies to measure social and economic impact due to the coronavirus pandemic in the US (10). The pulse survey contains information on demographic characteristics, education, income, employment, food sufficiency, access to healthcare physical and mental health and other COVID-19 related information such as vaccine and testing.

### Study design

We adopted a cross sectional and quasi-experimental design with mental health measurements in June 2020 and March 2021. Specifically, we used the survey results from Census Household Pulse Survey (HPS) during the following two waves: Week 7: June 11- June 16, 2020, and Week 27: March 17- 29, 2021. We chose these two waves of data for comparison purposes because in June 2020 individuals were subject to high stress due to prolonged health regulations, lock downs, and social isolation due to physical and social distancing, and in March 2021 vaccines were approved and became available to all adults over 18 years of age.

The inclusion criteria for the study were non-missing data on PHQ-2 and GAD-2 scores. Between week 7 and week 27, the census pulse survey consisted of 131,941 adults representing 209,245,170 adults in the United States.

### Measures

The dependent variables examined in this study were depression (yes/no) and anxiety (yes/no) based on Patient Health Questionnaire (PHQ-2) and Generalized Anxiety Disorder (GAD-2) questionnaires. PHQ-2, a patient-reported outcome measure (PROM) assesses depression symptoms with two questions. 1. Little interest or pleasure in doing things. 2. Feeling down, depressed, or hopeless. GAD-2 assesses anxiety symptoms with two questions. 1. Feeling nervous, anxious or on edge. 2. Not being able to stop or control worrying. Each question is rated from 0 to 3 (Not at all (0), several days (1), More than half the days(2), and nearly every day (3). Thus, both PHQ-2 and GAD-2 scores range from 0 to 6. Adults with PHQ-2 score 3 or greater should be screened for major depressive disorder (11). Sensitivity of PHQ-2 is 97% and specificity is 67%. Adults with GAD-2 score 3 or greater should be screened for generalized anxiety disorder (12). Sensitivity of GAD-2 is 86% and specificity is 83%. In our study, adults who scored 3 or greater PHQ-2 were classified as having depression and adults who scored 3 or more in GAD-2 were classified as having anxiety.

Other explanatory variables included age, sex, food insecurity, education, income, race and ethnicity, marital status, loss of employment (whether reported lost work during the past 4 weeks), region.

### Statistical analysis

We tested group differences using Rao-Scott chi-square. Multivariable logistic regressions were used to analyze factors associated with the presence of depression and anxiety respectively. In these regressions, our main focus was on time and we controlled for age, sex, food insecurity, education, income, race and ethnicity, marital status, loss of employment, and region. All analyses were conducted with the SAS survey procedures to take the survey weights provided by the Census pulse survey into consideration.

## Results

Table 1 illustrates the characteristics of adult respondents in week 7 and week 27 in US Census Pulse Survey. There were 51.8% females, 64.3% white, 10.7% African American, 16.2% Hispanic/Latino, 5.0% Asian and 3.8% other race or multiracial; and 9.6% did not have health insurance. Demographic, socio-economic, and healthcare access (age, sex, race and ethnicity, education, and health insurance) did not differ between June 2020 and March 2021 (Table 1).

**Table 1:** Description of selected characteristics of adult (18 years or older) respondents in weeks 7 (june 2020) and 27 (march 2021) United States Census Pulse Survey.

		**June 2020**		**March 2021**		**Chi-square**	***p*-value**
		**N**	**Wt. %**	**N**	**Wt. %**		
**ALL**		**68,009**	**100.0**	**63,932**	**100.0**		
**Sex**						0.085	0.770
	Female	40,588	51.6	37,828	52.0		
	Male	27,421	48.4	26,104	48.0		
**Race and ethnicity**						1.673	0.796
	White	52,049	63.5	48,968	65.4		
	African American	5,009	11.0	4,225	10.3		
	Latino/Hispanic	5,677	16.5	5,660	15.8		
	Asian	2,940	5.0	2,990	5.1		
	Other race	2,334	4.0	2,089	3.5		
**Marital status**						1.101	0.894
	Married	39,596	55.9	38,090	56.7		
	Widow	3,510	3.9	3,893	4.5		
	Sep/Div	11,859	13.9	10,937	13.7		
	Never married	12,813	26.0	10,634	24.6		
**Education**						1.929	0.587
	Less than high school	1,259	8.1	1,180	7.6		
	High School	21,806	51.7	19,983	49.3		
	Associate degree	6,961	8.9	6,638	10.0		
	College	37,983	31.3	36,131	33.1		
**Income**						14.451	0.071
	LT $25,000	6854	15.5	5645	12.7		
	$25,000–$34,999	5820	11.3	4894	9.7		
	$35,000–$49,999	7094	11.8	6297	10.8		
	$50,000–$74,999	11437	17.0	10433	16.5		
	$75,000–$99,999	9276	12.1	8462	11.7		
	$100,000–$149,999	11608	13.9	10996	14.0		
	$150,000–$199,999	5455	6.0	5452	6.4		
	GE $200,000	6319	6.3	6414	7.3		
**Region**						0.152	0.985
	Northeast	11,453	17.2	10,054	17.2		
	South	23,280	38.1	19,855	37.7		
	Midwest	14,141	20.4	13,172	20.7		
	West	19,135	24.2	20,851	24.3		
**Employment**						2.607	0.272
	Employed	38,862	54.7	37,135	59.1		
	Not employed	29,082	45.2	26,715	40.7		
**Health insurance**						1.266	0.737
	Private	52,992	71.0	49,944	73.2		
	Public	9,939	16.6	10,035	15.7		
	None	3,995	10.3	2,918	8.6		
**Lost work**						2.489	0.288
	Yes	26,204	47.5	22,539	43.8		
	No	41,711	52.4	41,284	56.1		
**Food sufficiency**						10.829	0.004
	Yes	46,016	58.4	48,773	67.9		
	No	21,803	41.2	15,020	31.8		

Based on adults (aged 18 or older) who responded to the United States Census Pulse Survey in week 7 or Week 27, with no missing data in Patient Health Questionnaire-2 and Generalized Anxiety Disorder 2-item questions. Due to missing data (marital status, employment, income, health insurance, lost work, food sufficiency), the column percentages may not add to 100%. Missing data are not included in the table. Group differences were tested with Rao-Scott chi-square statistics.

LT, Less than; GE, Greater than or equal; Sep/Div, Separated Divorced; Wt, Weighted. The red indicates that these are statistically significant.

With respect to depression, 25.0% in June 2020 and 24.6% in March 2021 had PHQ-2 score **≥** 3 (Table 2). With respect to anxiety, 31.7% in June 2020 and 30.0% in March 2021 had a GAD-2 score **≥** to 3 (Table 2). The differences were not statistically significant. In adjusted analysis, there were no significant differences in likelihood of depression and anxiety between June 2020 and March 2021.

**Table 2:** Description of selected characteristics of adult respondents by depression and anxiety (row percentages) United States Census Pulse Survey–week 7 (june 2020) and week 27 (march 2021).

		**With depression**				**With anxiety**			
		**N**	**Wt. %**	**Chi-square**	***p*-value**	**N**	**Wt. %**	**Chi-square**	***p*-value**
**ALL**	**26,903**	**24.8**			**36,484**	**30.9**		
**Time**				0.035	0.852			0.48	0.489
	June 2020	14,285	25.0			20,221	31.7		
	March 2021	12,618	24.6			16,263	30.0		
**Sex**				1.666	0.197			7.119	0.008
	Female	17,240	26.3			24,376	34.3		
	Male	9663	23.2			12,108	27.3		
**Race and ethnicity**				3.900	0.420			2.812	0.590
	White	19,229	23.2			26,662	29.6		
	African American	2,290	27.6			2,969	34.2		
	Latino/Hispanic	2,946	28.3			3,840	34.1		
	Asian	1,101	22.6			1,369	26.6		
	Other race	1,337	33.3			1,644	36.6		
**Marital status**				35.555	<0.001			25.033	<0.001
	Married	12,003	18.6			18,150	25.3		
	Widow	1,459	24.8			1,668	26.4		
	Sep/Divorced	6,105	30.2			7,584	35.8		
	Never married	7,203	35.8			8,915	41.4		
**Education**				12.739	0.005			6.895	0.075
	LT High School	835	31.8			966	37.7		
	High School	10,881	27.7			13,257	32.5		
	Associate degree	3,180	26.8			4,073	33.5		
	College	12,007	18.0			18,188	26.0		
**Income**				36.893	<0.001			34.120	<0.001
	LT $25,000	4,741	37.3			5,470	43.0		
	$25,000–$34,999	3,170	32.0			3,868	37.3		
	$35,000–$49,999	3,384	29.2			4,220	34.8		
	$50,000–$74,999	4,723	24.6			6,343	31.4		
	$75,000–$99,999	3,141	20.7			4,461	26.7		
	$100,000–$149,999	3,271	16.9			5,006	22.6		
	$150,000–$199,999	1,353	14.6			2,249	21.3		
	GE $200,000	1,353	13.2			2,388	19.6		
**Region**				0.833	0.842			0.880	0.830
	Northeast	4,149	23.5			5,945	28.1		
	South	9,218	25.8			12,230	35.1		
	Mid-west	5,178	23.4			7,053	43.5		
	West	8,358	25.5			11,256	37.4		
**Employment**				6.291	0.043			1.974	0.378
	Employed	14,169	21.9			20,931	41.0		
	Not employed	12,702	28.6			15,512	22.4		
**Health insurance**				16.766	<0.001			13.160	0.004
	Private	18,742	21.9			26,569	20.9		
	Public	5,113	29.9			6,182	48.1		
	None	2,531	37.8			3,041	25.9		
**Lost work**				45.427	<0.001			61.924	<0.001
	Lost work	14,147	33.2			18,832	41.0		
	No	12,715	17.8			17,594	22.4		
**Food sufficiency**				133.962	<0.001			89.638	<0.001
	Yes	12,523	15.4			18,803	20.9		
	No	14,309	40.9			17,592	48.1		
**Comorbid dep and anxiety**				496.000	<0.001			496.000	<0.001
	Yes	21610	65.7			21610	81.8		
	No	5293	6.5			14874	14.1		

Based on adults (aged 18 or older) who responded to the United States Census Pulse Survey in week 7 or Week 27, with no missing data in Patient Health Questionnaire-2 and Generalized Anxiety Disorder 2-item questions. Missing data (marital status, employment, income, health insurance, lost work, and food sufficiency) are not presented in the table. Group differences were tested with Rao-Scott chi-square statistics.

Dep, Depression; LT, Less than; GE, Greater than or equal; Sep/Div, Separated Divorced; Wt, Weighted. The red indicates that these are statistically significant.

In adjusted logistic regression (Table 3), those who reported food insufficiency (AOR = 2.93, 95% CI = 2.25, 3.79) and those who were never married (AOR = 1.52, 95% CI = 1.02, 2.28) were more likely to have depression compared to those who reported food sufficiency and were married. Adults who did not lose work during the pandemic were less likely to have depression (AOR = 0.62, 95% CI = 0.47, 0.83) compared to those who lost employment.

**Table 3:** Adjusted odds ratios and 95% (confidence intervals) selected characteristics from separate logistic regressions on depression and anxiety United States Census Pulse Survey–Week 7 (june 2020) and week 27 (march 2021).

		**AOR**	**95% CI**	***p*-value**	**AOR**	**95% CI**	***p*-value**
**Time**						
	June 2020(Ref)						
	March 2021	0.87	[0.65,1.16]	0.3495	0.94	[0.73, 1.20]	0.6060
**Sex**							
	Female	1.14	[0.85, 1.53]	0.3690	1.39	[1.06, 1.83]	0.0172
	Male (Ref)						
**Age**	0.93	[0.89, 0.97]	0.0005	0.91	[0.88, 0.95]	<0.001
**Race and ethnicity**						
	White (Ref)						
	AA	0.82	[0.50, 1.34]	0.4221	0.82	[0.50, 1.33]	0.4174
	Latino	0.74	[0.43, 1.26]	0.2629	0.70	[0.43, 1.13]	0.1408
	Asian	0.85	[0.40, 1.79]	0.6615	0.70	[0.37, 1.33]	0.2677
	Other race	1.11	[0.58, 2.14]	0.7424	0.91	[0.45, 1.83]	0.7844
**Marital status**						
	Married (Ref)						
	Widow	1.55	[0.78, 3.11]	0.2104	1.15	[0.56, 2.37]	0.6979
	Sep/Div	1.45	[1.00, 2.12]	0.0506	1.28	[0.88, 1.85]	0.1922
	Never Married	1.52	[1.02, 2.28]	0.0381	1.25	[0.85, 1.83]	0.2455
**Education**						
	LT HS	1.18	[0.55, 2.54]	0.6628	0.99	[0.52, 1.89]	0.9794
	HS	1.18	[0.86, 1.62]	0.3047	0.94	[0.72, 1.24]	0.6712
	Assoc deg	1.24	[0.75, 2.04]	0.4065	1.06	[0.69, 1.63]	0.7816
	College (Ref)						
**Income**						
	LT $25,000 (Ref)						
	$25,000–$34,999	0.98	[0.53, 1.81]	0.9355	0.95	[0.56, 1.62]	0.8593
	$35,000–$49,999	0.98	[0.58, 1.65]	0.9282	0.96	[0.53, 1.73]	0.8897
	$50,000–$74,999	0.87	[0.51, 1.49]	0.6096	0.90	[0.54, 1.51]	0.6906
	$75,000–$99,999	0.83	[0.44, 1.56]	0.5547	0.82	[0.47, 1.43]	0.4821
	$100,000–$149,999	0.76	[0.40, 1.45]	0.3986	0.74	[0.41, 1.31]	0.2970
	$150,000–$199,999	0.72	[0.36, 1.45]	0.3566	0.75	[0.40, 1.42]	0.3764
	GE $200,000	0.72	[0.35, 1.49]	0.3738	0.75	[0.38, 1.47]	0.3981
**Region**						
	Northeast (Ref)						
	South	1.06	[0.71, 1.57]	0.7806	1.05	[0.72, 1.53]	0.8087
	Mid-west	0.96	[0.60, 1.53]	0.8623	0.93	[0.60, 1.44]	0.7473
	West	1.04	[0.68, 1.61]	0.8491	1.07	[0.70, 1.62]	0.7625
**Health insurance**						
	Private (Ref)						
	Public	1.12	[0.77, 1.63]	0.5606	1.08	[0.73, 1.59]	0.6935
	None	1.14	[0.70, 1.88]	0.5965	1.10	[0.65, 1.85]	0.7215
**Lost work**						
	Lost work (Ref)						
	No	0.62	[0.47, 0.83]	0.0012	0.57	[0.44, 0.74]	<0.001
**Food sufficiency**						
	Yes (Ref)						
	No	2.92	[2.25, 3.79]	0.0000	2.82	[2.09, 3.81]	<0.001

Based on adults (aged 18 or older) who responded to the United States Census Pulse Survey in week 7 or Week 27, with no missing data in Patient Health Questionnaire-2 and Generalized Anxiety Disorder 2-item questions.

AOR, Adjusted Odds Ratio; 95% CI, 95% Confidence Interval; Dep, Depression; LT, Less than; GE, Greater than or equal; Sep/Div, Separated Divorced.

In adjusted logistic regression (Table 3), those who reported food insufficiency (AOR = 2.82, 95% CI = 2.09, 3.81), loss of employment (AOR = 0.57, 95% CI = 0.44, 0.74) were more likely to be anxious compared to those with food sufficiency and did not lose employment. Being married was associated with lower odds of anxiety (AOR = 1.25, 95% CI = 0.85, 1.83). Being a female was associated with higher odds of anxiety (AOR = 1.39, 95% CI = 1.06, 1.83) compared to males.

## Discussion

This study examined the association of COVID-19 vaccine availability and mental health. We observed adult depression prevalence rate at 25.0% in June 2020 and at 24.6% in March 2021 based on Census pulse survey. Therefore, the result suggests that the depression prevalence was relatively stable over this time period. We also report that anxiety was initially 31.7% at the beginning of the pandemic and 30.0% in March 2021. People who lost their jobs, had food insecurity, and were older were more likely to experience depression and anxiety in the study period. Females, in general, were more likely to experience anxiety, and people who were never married were more likely to experience depression during the study period.

These depression and anxiety rates were much higher than numbers found in the literature in the year preceding the COVID-19 pandemic. For example, one study found that in 2019, 18.5% of U.S. adults were experiencing depression, of which 11.5% reported mild symptoms, 4.2% reported moderate symptoms, and 2.8% reported severe symptoms (13). Another study showed that during that same year, there were 15.6% who reported experiencing the anxiety, of which 9.5% reported mild symptoms, 3.4% reported moderate symptoms, and 2.7% reported severe symptoms (14).

Several studies during the pandemic showed an increase in depression and anxiety symptoms that were above the 2019 levels and were similar to the results of this study. A meta-analysis with pooled prevalence showed depression levels at 25% from January 1, 2020 to May 8, 2020 (4). In another meta-analysis conducted without a lower time limit and until May 2020, the prevalence of depression was 33.7% (much higher than our results); however, the prevalence of anxiety was 33.7% (similar to our results) (5). Other researchers have also reported that there was a higher burden of depression symptoms among U.S. adults in a study from March 31, 2020, to April 13, 2020 in which 27.8% reported depression symptoms (15).

Our study differs from the previous ones in that its time frame encompasses the availability of the COVID-19 vaccine for the general public and the potential for some resolution of depression and anxiety. The initial high levels of depression and anxiety were not unexpected as COVID-19 brought uncertainty and stress with its high transmission and number of hospitalizations and deaths in the early months of 2020. Additionally, the poor health messaging, lockdowns, economic downturn, and poor management of the pandemic in early 2020 were also factors that could be expected to impact depression and anxiety symptoms. A prior study found that COVID-19 vaccination improved anxiety and depression in chronic kidney disease patients (9). However, in this study, depression and anxiety levels remained high in March 2021, despite the widespread availability of the vaccines that were shown to sharply decrease severe COVID-19, hospitalizations, and death (16).

Three main factors may explain the high levels of depression and anxiety that did not subside after the availability of vaccines: vaccine hesitancy, concern for children ineligible for the vaccine, and social determinants. There is significant vaccine hesitancy in the Us. One online survey indicated that 41% of participants reported a belief of an adverse effect on fertility with the vaccination, and 38% reported being unsure about an adverse effect on fertility (17). In the U.S., the mixed messages, political discourse, and social media were evident. In a study of social networking tweets, the most retweeted tweets had misinformation (18). The researchers suggested that many of the tweets were from anti-vaxxer activists and systematic professional sources (18). Prior studies that shown that individuals with less education, less income, and who were black were more likely to have vaccine hesitancy or decline vaccinations (19, 20). Another study found that children and adolescents in England who had prior COVID-19 infection were more hesitant to receive vaccine and also had lower level of depression and anxiety (21). It is interesting to note that in a study conducted in Germany, COVID-19-related anxiety was associated with higher vaccine acceptance (22).

The other potential factor for maintaining high levels of anxiety and depression symptoms was concern about children and COVID-19. In March 2021, children under 12 years did not have access to vaccinations, and school boards were considering returning the children to in-person learning. In a study conducted in mid-March 2020, parents of children from primary school to college were surveyed, and parents who perceived stress and had children in middle or high school were at greater risk for depression and anxiety (23).

Our findings also indicated an association of depression and anxiety with social determinants of health such as employment, food sufficiency, and marital status (a proxy for social support). Regardless of COVID-19, individuals with untreated depressive disorders had lower employment rates (24). In a study of 424 adults, employment at baseline was associated with lower depressive symptoms throughout the life course of the depression (25). In one study, from June 15 to June 30, 2020, direct or household employment loss (job insecurity) was associated with a greater risk of poor mental health (26). Food insecurity has also been an identified risk factor for depression in older adults (27, 28). Researchers conducting a meta-analysis for risk factors for depression and anxiety indicated a positive relationship with food insecurity (29).

In our study, we found that persons who had never married were associated with depressive symptoms. In a literature review of marriage and psychiatric illness prior to the pandemic, marriage was both a protecting and predisposing factor for psychiatric illness, depending upon the quality of the marriage (30). A study of job loss during the pandemic and marriage indicated that married individuals were 1–2% less likely to develop mental health problems related to work/income (31). Another study indicated that during the pandemic, the quality of the marriage was related to depression and anxiety and that individuals with no relationships scored better than individuals with poor ones (32).

Our finding that depression and anxiety symptoms did not improve after COVID-19 vaccine became available has implications for future mental healthcare needs and healthcare delivery. During the period studied in this research, there was increased use of telehealth for anxiety and depression in some settings (33). In one study, telehealth reduced depression but not anxiety during the pandemic (34). In a survey study conducted in Arkansas, 42% of participants reported using telehealth; and those with anxiety and/or depression had three times greater odds than those with no diagnosis (35). Telehealth may be a viable means by which to meet mental healthcare needs beyond COVID-19.

With each wave of COVID-19, there may continue to be high levels of depression and anxiety symptoms. The means to provide pharmacological and non-pharmacological therapies to alleviate the mental health burden need to be expanded. Future studies need to explore barriers to COVID-19 related mental healthcare utilization and the impact of mental health therapies on outcomes among adults with depression and or anxiety.

### Strengths and limitations

Our study has many strengths and some limitations. We used nationally representative data with near real-time collection. The findings from this study may inform public health planning and policies to address mental health. Availability of repeated cross-sections enabled assessment of COVID-19 related mental health burden over time. However, the survey lacked information on some variables such as chronic conditions, health status, loss/impact of COVID-19 on family and friends, the severity of depression and anxiety, physical activity, and vaccine hesitancy that may have influenced mental health.

## Conclusion

Depression and anxiety symptoms did not change significantly between June 2020 and March 2021. These results suggest that the effects of the pandemic on mental health continue to persist despite the widespread availability of vaccines that would have been considered to assuage some of the symptoms.

## Data availability statement

Publicly available datasets were analyzed in this study. This data can be found here: https://www.census.gov/programs-surveys/household-pulse-survey/datasets.html.

## Author contributions

CS and US contributed to conception and design of the study. LR and US performed the statistical analysis. All authors contributed to the writing of the manuscript, read, and approved the submitted version.

## Funding

This project described was supported in part by the National Institute of General Medical Sciences, 5U54GM104942-05 (RW) and by the National Institutes of Health (NIH) Agreement No. 1OT2OD032581-01 (US) and NIH/1OT2HL158258-01 (US), and the National Institute on Minority Health and Health Disparities through the Texas Center for Health Disparities (NIMHD), 5U54MD006882-10 (HW and US).

## Conflict of interest

The authors declare that the research was conducted in the absence of any commercial or financial relationships that could be construed as a potential conflict of interest.

## Publisher's note

All claims expressed in this article are solely those of the authors and do not necessarily represent those of their affiliated organizations, or those of the publisher, the editors and the reviewers. Any product that may be evaluated in this article, or claim that may be made by its manufacturer, is not guaranteed or endorsed by the publisher.

## Author disclaimer

The views and conclusions contained in this document are those of the authors and should not be interpreted as representing the official policies, either expressed or implied, of the NIH.
